# P01-01 Assessment of local governments’ involvement in sport and physical activity policy promotion – LoGoPAS project

**DOI:** 10.1093/eurpub/ckac095.001

**Published:** 2022-08-29

**Authors:** Petru Sandu, Razvan Mircea Chereches, Antonia Papiu, Peter Gelius, Karim Abu-Omar, Noriko Takeda, Yukio Oida, Tanja Onatsu, Katariina Tuunanen

**Affiliations:** 1 Public Health, University Babes-Bolyai, Cluj-Napoca, Romania; 2 Public Health, University of Medicine and Pharmacy Iuliu Hatieganu, Cluj-Napoca, Romania; 3 Department Sportwissenschaft und Sport, University of Erlangen-Nuremberg (Friedrich-Alexander-Universität Erlangen-Nürnberg), Erlangen, Germany; 4 Physical Activity and Health Promotion Studies, Center for Promotion of Higher Education, Kogakuin University, Tokyo, Japan; 5 School of Life Science and Technology, Chukyo University, Nagoya, Japan; 6 Fit for life program, LIKES – Research Centre for Physical Activity and Health, Jyväskylä, Finland

**Keywords:** local governments, local HEPA policy, promotion, evaluation, multi-country

## Abstract

**Background:**

The involvement of local governments in physical activity (PA) promotion represents a key factor to drive change at grassroots level, based on identified specific needs and solutions, tailored to each local context (needs, resources, etc.). The aim of LoGoPAS, Erasmus + Sport co-financed project (2020-2021), is to assess, promote and support local governments' involvement in PA (policy) promotion.

**Methods:**

A mixed methods approach has been put in place (including thematic document analysis of grey literature, semi-structured stakeholder interviews, group consensus methodologies) to explore and analyze the current legally binding and voluntary activities of local goverments in PA (policy) promotion. The study is being conducted at local level in Finland, France, Germany, Japan and Romania. Already validated instruments to evaluate local PA policy, such as L-PAT (Local Policy Audit Tool), Capla SANTE or TEAviisari will be used to harmonize data collected from each partner country, at local level.

**Results:**

Locally collected data from partner countries reveal significant differences in the organization and delivery of PA related policies with more regulations and structures in the western countries (e.g. Germany or Finland) and more opportunistic and mixed approach (related to the purpose of the PA related projects) in Romania. Also, the currently available instruments for evaluation of local PA policies (those used for data harmonization in our project - see methods section) can benefit from additions in order to capture the complexities of the engagement of local governments in PA promotion.

**Conclusion:**

Although the roles of local governments in PA promotion has been widely acknowledged (e.g. by WHO or the European Comission), there are currently few hands-on instruments for policy-makers and other actors at local level to advance PA promotion agenda and activities. Approaches tailored to local contexts may help engage local governments, while international networking may foster the exchange of experiences and help optimize interventions.

